# Neuroimmune Regulation of GABAergic Neurons Within the Ventral Tegmental Area During Withdrawal from Chronic Morphine

**DOI:** 10.1038/npp.2015.221

**Published:** 2015-08-12

**Authors:** Anna M W Taylor, Annie Castonguay, Atefeh Ghogha, Pia Vayssiere, Amynah A A Pradhan, Lihua Xue, Sadaf Mehrabani, Juli Wu, Pat Levitt, Mary C Olmstead, Yves De Koninck, Christopher J Evans, Catherine M Cahill

**Affiliations:** 1 Department of Anesthesiology and Perioperative Care, University of California, Irvine, CA, USA; 2 Hatos Center for Neuropharmacology, Semel Institute for Neuroscience and Human Behavior, University of California, Los Angeles, CA, USA; 3 Institut Universitaire en Santé Mentale de Québec, Québec, QC, Canada; 4 Department of Psychiatry and Neuroscience, Université Laval, Québec, QC, Canada; 5 Department of Biomedical and Molecular Sciences, Queen's University, Kingston, ON, Canada; 6 Children's Hospital Los Angeles and the Keck School of Medicine of the University of Southern California, Los Angeles, CA, USA; 7 Department of Psychology, Queen's University, Kingston, ON, Canada

## Abstract

Opioid dependence is accompanied by neuroplastic changes in reward circuitry leading to a negative affective state contributing to addictive behaviors and risk of relapse. The current study presents a neuroimmune mechanism through which chronic opioids disrupt the ventral tegmental area (VTA) dopaminergic circuitry that contributes to impaired reward behavior. Opioid dependence was induced in rodents by treatment with escalating doses of morphine. Microglial activation was observed in the VTA following spontaneous withdrawal from chronic morphine treatment. Opioid-induced microglial activation resulted in an increase in brain-derived neurotrophic factor (BDNF) expression and a reduction in the expression and function of the K^+^Cl^−^ co-transporter KCC2 within VTA GABAergic neurons. Inhibition of microglial activation or interfering with BDNF signaling prevented the loss of Cl^−^ extrusion capacity and restored the rewarding effects of cocaine in opioid-dependent animals. Consistent with a microglial-derived BDNF-induced disruption of reward, intra-VTA injection of BDNF or a KCC2 inhibitor resulted in a loss of cocaine-induced place preference in opioid-naïve animals. The loss of the extracellular Cl^−^ gradient undermines GABA_A_-mediated inhibition, and represents a mechanism by which chronic opioid treatments can result in blunted reward circuitry. This study directly implicates microglial-derived BDNF as a negative regulator of reward in opioid-dependent states, identifying new therapeutic targets for opiate addictive behaviors.

## INTRODUCTION

Prescription, diversion, and illicit use of opioid therapeutics have emerged as a major problem in recent years (Compton and Volkow, 2006). A hallmark of opioid addiction is the high rate of relapse, which represents a significant clinical challenge. The propensity for dependent opioid abusers to relapse has been attributed to the dysphoria associated with drug abstinence that disrupts the ability to process rewards (Weiss and Koob, 2001; Goeldner *et al*, 2011).

Reduced activity within the mesocorticolimbic dopamine (DA) system is one element that contributes to a negative affective state during withdrawal from drugs of abuse, such as opioids (Elman *et al*, 2013). This negative affect is proposed to contribute to pathological addictive behaviors (Koob and Le Moal, 2008). DA is the canonical neurotransmitter involved in motivated behavior and reward learning, and may contribute to the negative affective state during drug withdrawal. Striatal basal and evoked DA levels are significantly lower during withdrawal from chronic opioids (Acquas and Di Chiara, 1992; Zhang *et al*, 2009). Loss of striatal DA is linked to a deficit in spontaneous activity of DA neurons and failure of morphine to elicit the typical DA bursting activity following opioid withdrawal (Diana *et al*, 1995; Georges *et al*, 2006). Further evidence suggests that the loss of function of DA neurons in opioid-dependent states has broader implications for general reward processing. For example, microdialysis studies found cocaine-induced DA release to be significantly attenuated following chronic opioid exposure (Leri *et al*, 2003). This loss of cocaine-stimulated extracellular DA levels is reflected in a reduction of spontaneous and drug-precipitated cocaine-seeking behavior (Stevenson *et al*, 2004; Leri *et al*, 2006). Brain-derived neurotrophic factor (BDNF) has been identified as being a critical modulator of VTA DA neurons in opioid-dependent animals (Koo *et al*, 2012; Vargas-Perez *et al*, 2014). Together, these studies indicate that the BDNF-impaired DA activity in opioid-dependent animals may undermine the ability of the VTA to elicit reward behaviors.

The current study identifies a novel neuroimmune mechanism by which opioid dependence interferes with mesolimbic DA function. We show that manipulation of BDNF or KCC2 can restore reward processing following chronic opioids, presenting a novel mechanism for therapeutic intervention in treating addictive behaviors mediated by dysphoric states.

## MATERIALS AND METHODS

### Animals

Male C57Bl/6 J and GAD65-GFP on C57Bl/6 background mice (The Jackson Laboratory, Bar Harbor, ME), 8–9-weeks old at the beginning of experimentation were used, unless otherwise indicated. Male Sprague-Dawley rats (Charles River, QC) 8–9-weeks old were used in experiments with intra-cerebral drug administration, in order to assure precise administration of drug to the VTA. Animals were housed in groups of two to four until surgery, and kept on a 12-h light/dark cycle with food and water available *ad libitum.* All behavioral tests were performed during the light phase. All procedures were conducted in accordance with the National Institutes of Health Guide for the Care of Use of Laboratory Animals and were approved by the UCLA Institutional Animal Care and Use Committee.

### Drug Treatment

Mice were made opioid-dependent by twice-daily injections of escalating doses of morphine sulfate (10, 20, 30, 40 mg/kg, intraperitoneal (i.p.); National Institutes on Drug Abuse (NIDA), Bethesda, MD) for 4 days. Control animals (opioid-naïve) received twice-daily vehicle (saline, 0.9% NaCl, i.p.) injections in lieu of the drug. To examine the impact of reducing the morphine-induced microglial response, a separate group of mice received twice-daily injections of minocycline hydrochloride (30 mg/kg, i.p.; Sigma, St Louis, MO) or (+) naloxone (30 ng/kg, i.p.; NIDA) concomitant with morphine or saline beginning at the first morphine injection, until the end of the place preference protocol. Rats were made morphine-dependent by twice-daily injection of escalating doses of morphine sulfate (5, 8, 10, 10 mg/kg, i.p.) for 4 days (Cahill *et al*, 2007). A control group received twice-daily injections of saline (as above) in lieu of the drug. For all molecular studies, animals were killed 12 h after last morphine or saline injection (Day 5) or immediately after the post-conditioning day (Day 14).

### Conditioned Place Preference (CPP)

#### Systemic microglial inhibitors

**Figure 1:**
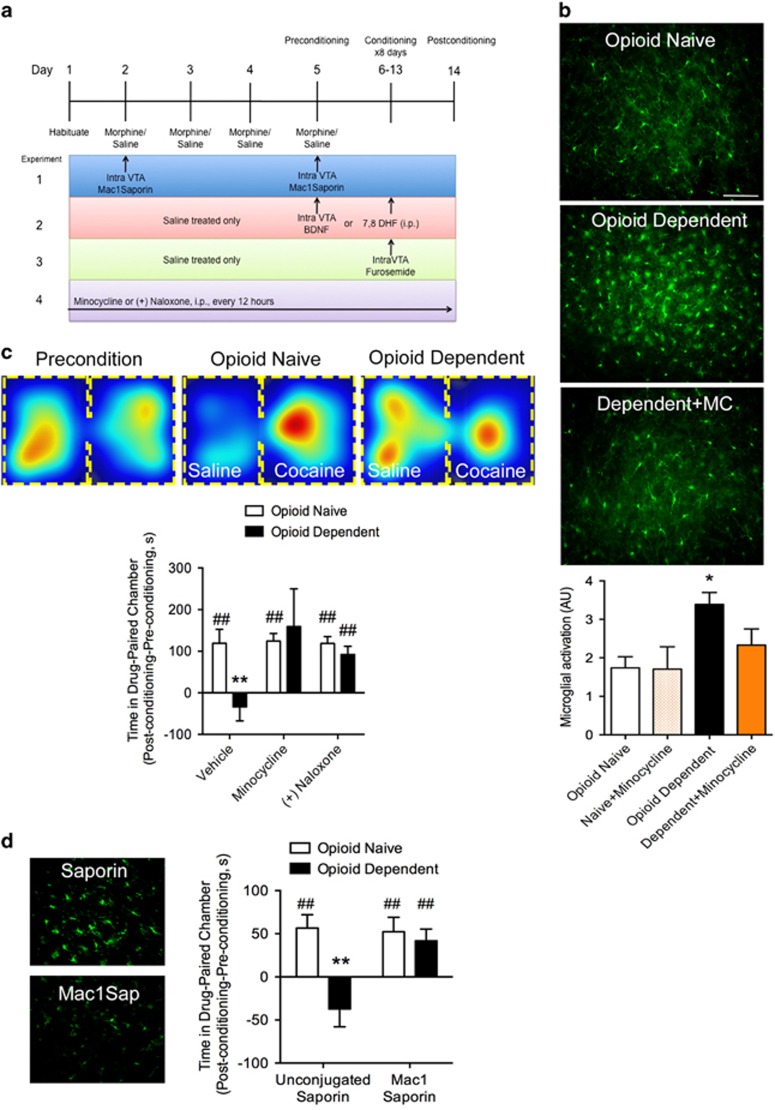
Chronic opioid treatment activates microglia and interferes with cocaine place preference. (a) Experimental design. (b) Microglia, identified by an antibody against IBA-1, were labeled in the VTA. Microglial activation was quantified based on defined morphological criteria, including cell body size and ramified branching. A chronic, ascending dose regimen of morphine resulted in significant microglial activation that could be blocked by daily systemic administration of the microglial inhibitor, minocycline (MC). Scale bar =100 uM. Bar graph represents quantification of activated microglia in VTA sections. *n*=4–5. Error bars=SEM, *p<0.05, *n*=3–12. (c) Top images display a heat map of total time spent in either the drug-paired or saline-paired chamber before (precondition) and after training. Bottom histograms plot the difference in time spent on the drug-paired side on the pre-conditioning day and the post-conditioning day. Opioid-dependent animals did not show a place preference to the cocaine-paired chamber. Systemic minocycline or (+)-naloxone, the opioid-inactive enantiomer previously shown to inhibit microglial activation, treatment recovered a cocaine place preference in opioid-dependent animals, but had no effect on cocaine place preference in opioid-naïve animals. Error bars=SEM, **p<0.01 when compared with opioid-naïve, ##*p*<0.01 when compared with baseline, *n*=5–10. (d) Animals in which VTA microglia were depleted with Mac-1-Saporin retained cocaine place preference in opioid-dependent animals. Mac-1-Saporin did not affect the development of cocaine place preference in naïve animals. Injection of unconjugated saporin had no effect on the development of cocaine place preference in naïve animals and it did not restore cocaine place preference in opioid-dependent animals. Error bars=SEM, **p<0.01 when compared with opioid-naïve, ##*p*<0.01 when compared with baseline, *n*=8–10. Photomicrographs depict IBA-1 labeling in VTA sections from animals treated with unconjugated saporin or Mac-1-Saporin. Scale bar=100 uM.

The mouse CPP was conducted using an unbiased, counter balanced, two-chamber apparatus (Supplementary Materials and Methods). Conditioning sessions consisted of mice receiving four trials of cocaine hydrochloride (10 mg/kg, i.p. NIDA) and four trials of saline, and were confined to the chambers for 15 min. The CPP assay began 12 h after the last morphine injection, and hence, all conditioning sessions were completed when animals were in a state of spontaneous opioid withdrawal (Figure 1a). On the post-conditioning day, animals were allowed free access to both chambers in a drug-free state, and the time spent in the drug-paired chamber was measured over 15 min. A preference score for each animal was calculated by taking the difference in time spent on the drug-paired chamber between the pre-conditioning and post-conditioning day.

#### Intra-VTA Mac-1-Saporin

The effect of specifically ablating microglia in the VTA on cocaine place preference in opioid-naïve and -dependent animals was assessed. Rats were implanted with a bilateral cannula targeting the VTA (mm from bregma): AP −6.30, ML +/−0.8, DV −8.0. Immediately prior to chronic opioid or saline treatment, animals were injected with Mac-1-Saporin or unconjugated saporin (ATSBio; 0.25 ug/0.5 ul per side). Animals were treated once again with Mac-1-Saporin or saporin immediately prior to the last injection of morphine. Twelve hours after the last opioid treatment, rats were conditioned to cocaine (10 mg/kg, i.p., as described above).

#### Intra-VTA furosemide

Rats were implanted with a bilateral intra-VTA cannula, as described above. Seven days after surgery, animals were treated with either furosemide (Sigma, 0.5 ul of a 1 mM in 0.5 ml 0.1% DMSO in saline) or vehicle over 3 min and then immediately conditioned to cocaine or saline, as described above. Furosemide was injected prior to each conditioning session over 8 days. On the final day, animals were allowed free access to both conditioning chambers, and the time spent on the drug-paired side was assessed. *Post hoc* analysis of slices counterstained with cresyl violet was carried out to verify correct injection sites and to confirm that repeated injections did not lead to significant damage of the VTA.

#### Intra-VTA BDNF and systemic 7,8-dihydroxyflavone

Rats were implanted with a bilateral intra-VTA cannula, as described above. Seven days after surgery, the effect of intra-VTA BDNF on cocaine CPP was assessed. In the BDNF group, BDNF (Sigma Aldrich; 25 ug in 0.5 ul saline) was injected into the VTA, over 3 min. A separate vehicle group received saline injections of the same volume. One day after BDNF or saline injection, cocaine (10 mg/kg, i.p.) place preference was run, as described above.

Opioid-naïve rats received systemic 7,8-dihydroxyflavone injections (Tocris Bioscience, Bristol, UK, 10 mg/kg, i.p.), a potent trkB agonist, 1 h prior to cocaine conditioning sessions. Control animals received vehicle (saline, i.p.) injections prior to cocaine conditioning sessions.

### Immunoblot Assay

VTA brain punches from opioid-naïve and -dependent mice treated with or without minocycline were assayed for BDNF protein content using western blot (Supplementary Material and Methods). Equal amounts of protein (25 ug) were loaded onto pre-cast 10% polyacrylamide gels (Bio-Rad, Hercules, CA) and transferred to nitrocellulose membranes. Membranes were incubated with an antibody against BDNF (1:150; Millipore, Billerica, MA) and visualized with a goat anti-rabbit antibody conjugated to a horseradish peroxidase (HRP; Jackson Immunoresearch, West Grove, PA). Results were analyzed with a computer-assisted densitometry analysis (Image J Software, NIH).

### Immunocytochemistry

Brain slices of the VTA were obtained from opioid-naïve and -dependent mice with or without minocycline treatment and prepared for immunocytochemical detection (Supplementary Material and Methods). For microglial staining, sections were incubated overnight with an antibody against IBA-1 (1:2000; Wako, Richmond, VA) at 4 °C followed by a goat anti-rabbit secondary antibody conjugated to Alexa Fluor 488 (1:1000, Millipore). For KCC2 staining, sections were incubated overnight with a rabbit antibody against KCC2 (1:500; Millipore) at 4 °C followed by a highly cross-adsorbed donkey anti-rabbit IgG conjugated to Alexa Fluor 594 (1:500; Invitrogen, Grand Island, NY). For KCC2 quantification, fluorescence intensity (total intensity per region of interest) was measured with Image J Software. For IBA1 quantification, the degree of microglial activation in the VTA was measured using a semi-quantitative method, based on defined morphological criteria, including cell body size, number of processes, and increasingly ramified morphology (Brettschneider *et al*, 2012). The level of microglial activation was scored on a linear scale (0–4) ranging from resting microglia (0) to highly activated (4).

### Fluorescent *In situ* Hybridization

Opioid-naïve and -dependent mice were prepared for *in situ* labeling. Sections containing the VTA were labelled with triple fluorescent *in situ* hybridization using RNAscope (Advanced Cell Diagnostics, Hayward, CA, USA) following the manufacturer's protocol. Probes against BDNF, TH, GAD, and integrin alpha M were used to label BDNF, DA neurons, GABAergic neurons, and microglia, respectively. Fluorescence signal intensity for each probe was quantified using ImageJ Software.

### Fluorescence Lifetime Imaging Microscopy

Three-hundred-micrometer-thick coronal brain sections corresponding to the VTA of C57Bl/6 and GAD65-GFP knock-in mice were incubated in artificial cerebral spinal fluid (126 NaCl, 2.5 KCl, 2 CaCl_2_, 2 MgCl_2_, 10 glucose, 26 NaHCO_3_, 1.25 NaH_2_PO_4_) containing 5 mM of the Cl^−^ indicator N-6-methoxyquinolinium acetoethylester (MQAE; Invitrogen) for 30–40 min at 34 °C. MQAE fluorescence was excited using a Zeiss LSM510 laser-scanning microscope coupled to a femtosecond-pulsed Ti-Sapphire laser (Chameleon Ultra, Coherent, Santa Clara, CA) tuned at 750 nm (Gagnon *et al*, 2013). After a control period of 50 s, the perfusion solution was switched to artificial cerebral spinal fluid containing 15 mM KCl (osmolarity adjusted using mannitol) to reverse KCC2-mediated Cl^−^ transport to force Cl^−^ accumulation inside the cell leading to a quenching of the measured lifetime (Chorin *et al*, 2011). Briefly, following the results of previous studies (Digman *et al*, 2008), we converted the photon timing histograms of each acquired lifetime image to phazer plots. To compare between groups, the average lifetime slope corresponding to the peak change in MQAE fluorescence was determined and compared. Means were calculated for each group (*n*=6–9 animals) for analysis.

### Statistics

For CPP, groups were compared with a Student's unpaired t-test or two-way ANOVA followed by *post hoc* analysis, where appropriate. A one-sample t-test was also run to determine whether the change in time spent in the drug-paired chamber was greater than baseline precondition times in the drug-assigned chamber (0). For the immunoblot assay, groups were compared with a Kruskal-Wallis test followed by a Dunn's *post hoc* analysis. For all other assays, groups were compared with a Student's unpaired t-test or one-way ANOVA followed by *post hoc* analysis, where appropriate. Results were considered statistically significant when *p*<0.05.

## RESULTS

### Activated Microglia Mediate the Blunted Cocaine Reward in an Opioid-Dependent State

We first identified cellular changes in the VTA following 4-day, twice-daily injection of escalating doses of morphine and spontaneous withdrawal. On the basis of IBA-1 immunostaining, we found microglia exhibited significant activation in the VTA, as measured by morphological analysis (F_2,18_=5.49, *p*=0.01, *n*=3–12; Figure 1b). Microglia exhibited a stereotypical ‘activated' phenotype, including an enlarged, compact cell body, thickening or hyper-ramification of cell processes, and greater number of immunolabeled cells compared with saline-treated animals. Opioid-induced microglial activation was attenuated by concomitant treatment with the microglial inhibitor minocycline. The morphology of microglia observed in animals treated with both chronic morphine and minocycline were indistinguishable from the ramified ‘surveying' microglia observed in non-opioid-treated animals. Minocycline treatment in opioid-naïve animals did not produce any detectable changes in microglial morphology revealed by IBA-1 immunostaining.

To probe the function of the reward system following chronic morphine administration, we used cocaine reward, given the confounds of using an opioid to probe reward function in an opioid-receptor-desensitized state and that an opioid will alleviate withdrawal and could produce a negative reinforcement to drive reward-related behaviors. Blunted rewarding properties of cocaine have been observed previously following chronic morphine regimens (Stevenson *et al*, 2004; Leri *et al*, 2006), and we questioned whether microglia could play a role. In opioid-naïve animals, cocaine (10 mg/kg) induced a robust place preference, but, consistent with prior studies, failed to elicit a place preference in animals in an opioid-dependent state (F_interaction_(2,36)=3.41, *p*=0.04, F_microglial inhibitor_(2,36)=3.67, p=0.04, F_opioid treatment_(1,36)=2.38, *p*=0.13, *n*=5–10; Figure 1c). We next tested the effect of inhibiting microglial activation on cocaine place preference in opioid-dependent mice. We used two different microglial inhibitors, minocycline and the inactive enantionmer opioid antagonist (+)-naloxone. (+)-Naloxone does not bind opioid receptors, but has been shown to specifically inhibit microglial activation (Iijima *et al*, 1978; Lewis *et al*, 2012; Wang *et al*, 2012; Mattioli *et al*, 2014). Remarkably, inhibiting microglia throughout the entire experimental period restored cocaine place preference in opioid-dependent animals (Figure 1c). In contrast, inhibition of microglial activation had no effect on cocaine preference in opioid-naïve animals.

We next examined whether microglia specifically within the VTA are required for the loss of cocaine place preference in opioid-dependent animals. Opioid-naïve and -dependent rats received microinjections of Mac-1-Saporin directly into the VTA to selectively destroy microglia within this region. Mac-1-Saporin microinjection restored cocaine place preference in opioid-dependent animals, and did not affect cocaine place preference in opioid-naïve animals (F_interaction_(1,29)=5.92, *p*=0.02, F_microglial inhibitor_(1,29)=4.79, *p*=0.04, F_opioid treatment_(1,29)=9.29, *p*=0.005, *n*=8–10; Figure 1d). Control injection of unconjugated saporin did not affect cocaine place preference in either naïve or dependent animals (Figure 1d). Intra-VTA injection of Mac-1-Saporin or systemic treatment of opioids did not affect the amount of time spent in the neutral chamber on the post-conditioning day (average time in the neutral chamber was 261±29 s with no significant difference detected between groups).

Cocaine place preference is partially reliant on functional mu opioid receptors (Houdi *et al*, 1989; Kosten *et al*, 1991; Bilsky *et al*, 1992; Gerrits *et al*, 1995; Kuzmin *et al*, 1997). Because chronic morphine administration leads to the desensitization of mu opioid receptors (Williams *et al*, 2013), we tested whether microglial inhibition restored cocaine place preference via changes in mu opioid receptor function. Chronic opioid exposure led to significant reduction in E_max_ of [D-Ala2, N-MePhe4, Gly-ol]-enkephalin (DAMGO)-stimulated [^35^S]GTPγS binding in the VTA, indicative of decreased functional mu opioid receptors within this region (F(2,12)=12.9, *p*=0.001, *n*=4–6; Supplementary Figure S1). Concomitant treatment of minocycline with escalating doses of morphine did not recover DAMGO-stimulated [^35^S]GTPγS binding, showing that the drug does not impact the general desensitization of mu opioid receptors following chronic morphine exposure.

### Activated Microglia in Opioid-Withdrawn Animals Release BDNF that Contributes to Blunted CPP

We next performed a series of studies to elucidate the mechanism by which VTA-activated microglia contributes to blunted DA-mediated reward. We have previously reported that activated microglia release BDNF in the spinal cord after chronic morphine treatment (Ferrini *et al*, 2013). Together with the finding that BDNF serves as a negative modulator of opioid reward in the VTA (Koo *et al*, 2012), studies were performed to determine whether activated microglia-derived BDNF in the VTA mediates the blunted cocaine place preference in opioid-dependent animals.

**Figure 2:**
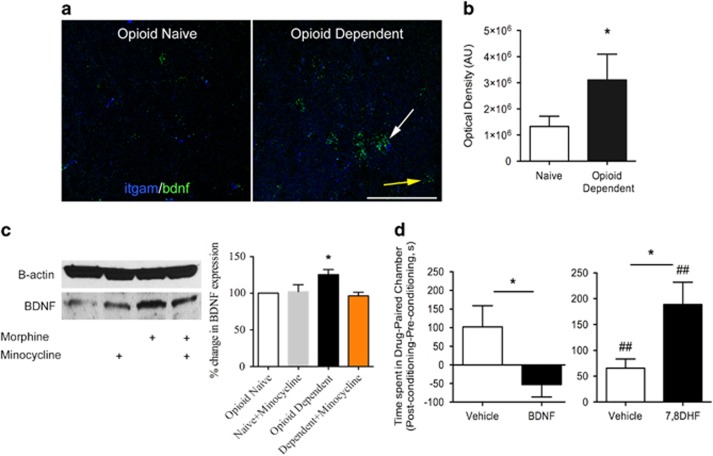
Chronic opioid treatment stimulates BDNF expression in the VTA that contributes to a blunted cocaine place preference. (a) BDNF mRNA (green) was detected with fluorescent *in situ* hybridization in both microglial (blue, identified with itgam promoter; white arrow) and non-microglial cells (yellow arrow). Chronic morphine caused an increase in BDNF transcripts in the VTA sections from opioid-dependent animals. (b) Total BDNF mRNA in VTA sections was semi-quantified using image analysis (see methods), and BDNF levels were significantly elevated in the VTA of opioid-dependent animals. Error bars=SEM, *p<0.05, *n*=4. (c) Tissue punches from the VTA were analyzed for BDNF protein expression using western blot analysis. Opioid-dependent animals showed an increase in BDNF protein (normalized to beta-actin) that was significantly greater than opioid-naïve brains. Minocycline reduced BDNF expression in the VTA of opioid-dependent animals. Error bars=SEM, *p<0.05, *n*=5. (d) Bilateral infusion of BDNF into the VTA of opioid-naïve animals prior to cocaine conditioning blocked the expression of cocaine place preference. Systemic injection with a potent trkB agonist, 7,8-dihydroxyflavone, enhanced cocaine CPP in opioid-naïve animals, compared with animals treated with vehicle. Error bars=SEM, *p<0.05 when compared with vehicle, ##*p*<0.01, when compared with baseline, *n*=6–8.

Using fluorescent *in situ* hybridization analysis (RNAscope), BDNF mRNA levels were significantly elevated in the VTA of opioid-dependent animals (t(6)=7.69, *p*=0.03, *n*=4; Figure 2a). Increased BDNF message was observed in microglia identified by co-expression of the integrin alpha M gene (itgam) (Figure 2a and b). Smaller increases in BDNF were also observed in TH+ cells, but not in GABAergic neurons (Supplementary Figure S2). BDNF protein was significantly elevated in the VTA of animals treated with chronic opioids (KW=14.92, *p*=0.02, *n*=5, Figure 2c). Pretreatment with minocycline blocked the rise in BDNF protein. These results suggest that although BDNF may be elevated in both neuronal and glial populations, inhibiting microglial activation in opioid-dependent animals is sufficient to reduce BDNF levels to resting levels.

We next examined whether BDNF was sufficient to block cocaine place preference in naïve animals. Opioid-naïve animals injected with BDNF directly into the VTA prior to place conditioning blocked the expression of cocaine place preference (t(8)=2.34, *p*=0.04, *n*=6–7; Figure 2d). Intra-VTA injection of BDNF did not affect the time spent in the neutral chamber on the post-conditioning day (Saline: 177.7±30.1 s *vs* BDNF: 218.7±26.1 s). To demonstrate the VTA-specific effect of BDNF-induced blunting of place preference, naïve animals were treated systemically with the TrkB agonist, 7,8-dihydroxyflavone. Treatment with a systemic TrkB agonist produced an increase in the magnitude of cocaine place preference (t(13)=2.51, *p*=0.03, *n*=7–8; Figure 2d), an opposite effect to that of local VTA administration of BDNF. These results support the finding that BDNF arising via microglial activation in the VTA of opioid-dependent animals is sufficient to block cocaine place preference.

### Withdrawal from Chronic Opioids Leads to Impaired Cl^−^ Transport Associated with a Loss of KCC2 Expression in VTA GABAergic Neurons

**Figure 3:**
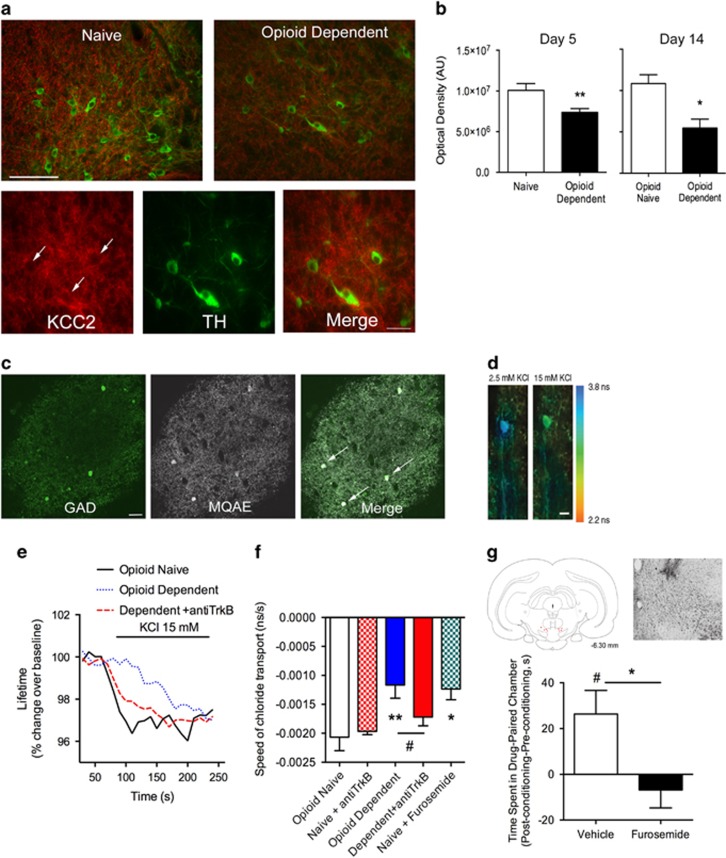
Decreased Cl^−^ transport in GABAergic VTA neurons related to a loss KCC2 expression. (a) Top images show KCC2 expression (red) surrounding tyrosine hydroxylase (TH)-positive neurons (green) in the VTA is significantly decreased after chronic morphine exposure (opioid-dependent). Images were taken at × 20 magnification. Scale =75 μm. Bottom images show KCC2 expression mainly found in non-TH+ neurons. White arrowhead indicates region of TH+ neuron with low KCC2 expression. Images were taken at × 40 magnification. Scale=50 μm. (b) Histogram depicts quantification of KCC2 labeling in the VTA of opioid-naïve and -dependent animals either 12 h after final morphine injection (Day 5) or after cocaine conditioned place preference (CPP) (Day 14). Opioid-dependent animals showed a significant reduction in KCC2 expression in the VTA. Error bars=SEM, *p<0.05, **p<0.01, *n*=9–12. (c) VTA slices from control animal showing MQAE (white) loaded exclusively in GAD+ neurons (green). White arrowheads indicate GAD+ neurons loaded with MQAE. Scale=20 μm. (d) Pseudocolor images showing lifetime maps from control VTA slices in the presence of 2.5 or 15 mM KCl^−^. (e) Representative MQAE lifetime plots. VTA slices from opioid-dependent animals showed slower rate of Cl^−^ transport into the cell, as measured by time to quench fluorescence of the Cl^−^ indicator, MQAE after an abrupt chance in extracellular K+ to reverse Cl^−^ transport. Acute treatment with the TrkB antagonist restored Cl^−^ transport. (f) Average slope at the 50-s time point for all groups. Movement of Cl^−^ into the cell was confirmed to be through KCC2 by application of a KCC2 antagonist, furosemide, which attenuated the change in MQAE fluorescence lifetime. *n*=7–9, error bars=SEM *p<0.05, **p<0.01, compared with opioid-naïve group and #*p*<0.05, compared with opioid-dependent group, *n*=7–9. (g) The KCC2 inhibitor, furosemide, injected directly into the VTA prior to cocaine conditioning, blocked cocaine CPP in opioid-naïve animals. Error bars=SEM, *p<0.05 compared with vehicle, #*p*<0.05 when compared with baseline, *n*=6–8. Left inset indicates injection sites (6.30 mm posterior to bregma) and right inset is a representative image of an injection site counterstained with cresyl violet (× 5 magnification).

**Figure 4:**
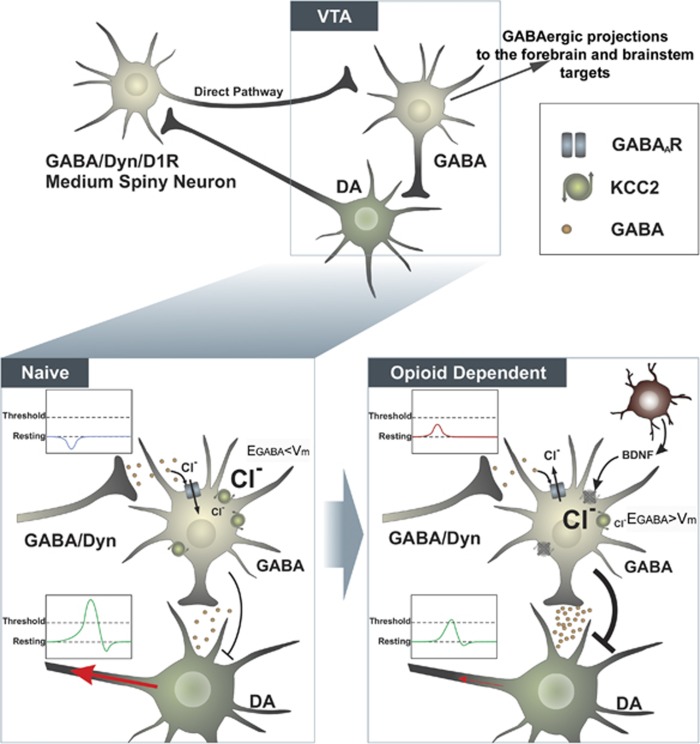
Normally, cocaine-stimulated DA in NAc activates reciprocal GABAergic medium spiny neurons (MSN) that project onto GABAergic VTA neurons. While GABAergic VTA neurons project to a range of forebrain and brainstem targets, cocaine mediated inhibition of GABAergic interneurons causes a net increase in VTA DA activity and further stimulates the release of DA in the NAc. In opioid-dependent states, KCC2 expression in GABAergic neurons of the VTA is mediated by BDNF. Decreased KCC2 expression leads to a dysregulation of the Cl^−^ gradient, compromising the inhibitory potential of these neurons. Release of GABA from the MSN projection neurons is less inhibitory on these neurons, leading to an increased excitability of GABAergic VTA neurons, and increased inhibition of DA neurons. Cocaine is no longer able to stimulate DA release in the NAc, and CPP is significantly impaired.

We next explored the mechanism by which BDNF may modulate VTA neuronal activity in opioid-dependent states. In the adult hippocampus and spinal cord, BDNF causes a downregulation of the K^+^/Cl^−^ co-transporter, KCC2 (Rivera *et al*, 2002; Coull *et al*, 2005; Ferrini *et al*, 2013). KCC2 is pivotal in maintaining the Cl^−^ gradient to enable GABA_A_-mediated hyperpolarization (Fiumelli *et al*, 2005). A previous study found that an acute opioid challenge in opioid-dependent animals potentiated, rather than inhibited, GABAergic currents in VTA GABAergic neurons, suggesting BDNF may have a specific effect on GABA_A_ function (Madhavan *et al*, 2010). We first examined changes in KCC2 expression in the VTA of opioid-dependent animals. Quantitative analysis of KCC2 immunolabeling in the VTA of opioid-naïve and -dependent tissue confirmed KCC2 downregulation in opioid-dependent states when measured 12 h after final morphine injection (day 5) (t(22)=2.87, *p*=0.009, *n*=9–12; Figure 3b). KCC2 expression remained low throughout the cocaine CPP in opioid-dependent animals (Day 14). Further, KCC2 expression was found exclusively on non-TH positive neurons (Figure 3a). We next investigated changes in Cl^−^ transport rate in VTA GABAergic neurons opioid-dependent states. The rate of Cl^−^ transport was measured using a fluorescent Cl^−^ indicator, MQAE, and a reverse transport strategy using abrupt elevation of extracellular K^+^. We have used this method previously to measure Cl^−^ transport rate within spinal cord neurons (Doyon *et al*, 2011; Ferrini *et al*, 2013). The MQAE dye loaded exclusively in GABAergic neurons, identified by GAD co-labeling in sections taken from transgenic GAD65-GFP mice (Figure 3c). We found that opioid-dependent animals in a state of withdrawal displayed a significantly slower rate of Cl^−^ transport (F(3,24)=0.25, *p*=0.02, *n*=7–9; Figure 3e and f). Application of a TrkB function-blocking antibody directly onto the VTA slices harvested from morphine-dependent animals restored normal Cl^−^ transport rate. These data show that BDNF has a critical role in mediating the chronic opioid-induced dysregulation of Cl^−^ homeostasis in VTA GABAergic neurons. Application of a KCC2 inhibitor, furosemide, to naïve slices inhibited Cl^−^ transport, reminiscent of the opioid-dependent state. Importantly, intra-VTA injection of furosemide blocked cocaine CPP in opioid-naïve animals (t(12)=2.41, *p*=0.03, *n*=6–8, Figure 3g). Furosemide treatment did not affect the time spent in the neutral compartment on the post-conditioning day (Saline: 352.5±39.1 s *vs* Furosemide: 391.4±43.4 s). This directly implicates the loss of KCC2 function in the VTA in driving blunted cocaine reward (Figure 4).

## DISCUSSION

### Increased VTA GABAergic Activity in Opioid-Dependent States

The present study provides the first evidence that microglia-derived BDNF can mediate the blunted DA activity in opioid-dependent and withdrawn states. Here, we show chronic opioid treatment causes dysregulation in transmembrane Cl^−^ homeostasis in GABAergic neurons of the VTA driven by BDNF and activated microglia.

DA neuronal activity is tonically inhibited by GABAergic VTA interneurons and extrinsic GABAergic projections. Activation of GABA_A_ receptors hyperpolarizes these inhibitory neurons and stimulates DA release (disinhibition). KCC2 normally transports Cl^−^ out of GABAergic neurons to maintain a low intracellular concentration and is critical for maintaining the inhibitory potential of GABA_A_ receptors (Rivera *et al*, 2002; Viitanen *et al*, 2010). While both DA and GABAergic neurons of the VTA express GABA_A_ receptors (Churchill *et al*, 1992; Kalivas, 1993), DA cell bodies do not express immunoreactive or functional KCC2, and appear to use alternative Cl^−^ transporters, such as Na+-dependent anion exchanger, to maintain the Cl^−^ gradient (Gulacsi *et al*, 2003). Therefore, downregulation of KCC2 in the VTA will disrupt GABA_A_ signaling in GABAergic rather than DA neurons. In the present study, GABAergic neurons of opioid-dependent animals exhibit a compromised Cl^−^ transport activity paralleled by a loss of KCC2 expression. Further, our data demonstrate that directly inhibiting KCC2 transport in naïve animals mimics the opioid-dependent state by blocking VTA Cl^−^ transport and cocaine CPP. Loss of KCC2 function leads to an accumulation of intracellular Cl^−^ within the cell and a reduction in Cl^−^ influx upon GABA_A_ receptor activation, resulting in a reduction in GABA-mediated hyperpolarization (Rivera *et al*, 1999; Hubner *et al*, 2001; Coull *et al*, 2003; Laviolette and van der Kooy, 2004; Taylor *et al*, 2015). These data support previous studies demonstrating that KCC2 inhibition compromises the inhibitory effect of a GABA_A_ agonist in the VTA (Ting *et al*, 2013). Our model predicts that decreased GABA_A_-mediated inhibition onto GABAergic neurons would increase inhibitory tone onto VTA DA neurons in opioid-dependent states. Decreased DA neuronal activity in opioid-dependent states is a robust finding in multiple studies using electrophysiological, neurochemical, and behavioral approaches (Leri *et al*, 2003, 2006; Stevenson *et al*, 2004; Georges *et al*, 2006). A shift in GABA_A_-mediated inhibition on GABAergic neurons is also supported by the observation that intra-VTA DAMGO potentiated, rather than inhibited, GABAergic currents on DA neurons in opioid-dependent animals (Madhavan *et al*, 2010). Further, the amplitude and frequency of spontaneous miniature GABA_A_ inhibitory postsynaptic potentials recorded from DA cells were greater in opioid-dependent animals in acute withdrawal, suggestive of increased GABA release onto DA neurons in opioid-dependent animals (Bonci and Williams, 1997).

In addition to the GABAergic interneurons, VTA GABAergic neurons project to a wide range of targets outside the midbrain, including the prefrontal cortex, hypothalamus, and habenula (Carr and Sesack, 2000; Taylor *et al*, 2014). Our current study cannot differentiate between these GABAergic populations, and it remains to be seen whether other behavioral phenotypes driven by these GABAergic projection neurons are also disrupted in opioid-dependent states.

### Changes in VTA GABAergic Activity is Mediated by BDNF and Activated Microglia

Activated microglia are known to modify opioid function. Chronic opioids activate glia in the spinal cord and blocking microglial activation prolongs the effectiveness of morphine analgesia and prevents the development of opioid-induced hyperalgesia (Raghavendra *et al*, 2004; Cui *et al*, 2006; Hutchinson *et al*, 2009; Ferrini *et al*, 2013). Activated microglia release BDNF, which acts as a modulator of neuronal activity. Release of BDNF from activated microglia in the spinal cord also results in a loss of GABAergic inhibition relating to a dysregulation of the transmembrane Cl^−^ gradient (Ferrini *et al*, 2013). There is also evidence that activated microglia contribute to supraspinal opioid modulation. Chronic opioid exposure results in activated microglia in supraspinal regions, such as the nucleus accumbens and VTA (Hutchinson *et al*, 2009). Blocking microglial activation suppresses morphine-induced respiratory depression and some measures of morphine-induced reward (Hutchinson *et al*, 2008). The current study directly implicates activated microglia in the VTA driving aberrant cocaine reward behavior in opioid-dependent states by interfering with GABAergic inhibition via the BDNF-KCC2 pathway. We have also shown that inhibition of microglial activation, either systemically or directly within the VTA, is sufficient to restore cocaine reward behavior in opioid-dependent animals (Figure 1).

VTA neurons express BDNF and TrkB receptors (Gall *et al*, 1992; Seroogy *et al*, 1994), and several studies suggest BDNF acts as a critical modulator of DA circuitry within the VTA. For example, injecting BDNF into the VTA decreases morphine place preference and blocks morphine-stimulated burst firing of DA neurons (Koo *et al*, 2012). In the same study, localized knockdown of BDNF in the VTA enhances the acute rewarding effect of morphine. Evidence that BDNF mediates the impaired DA signaling in opioid dependence includes injection of BDNF into the VTA, which mimicked an opioid-dependent reward-like state (Vargas-Perez *et al*, 2009). In our current study, we have shown that chronic morphine leads to increased total VTA BDNF protein levels as well as increased BDNF mRNA levels in VTA microglia. Selective injection of BDNF into the VTA blocked cocaine reward in opioid-dependent animals. These results support and build upon previously published work that suggests VTA BDNF acts as a negative modulator of drug reward (Koo *et al*, 2012). However, prior studies have shown hippocampal BDNF to facilitate learning and memory in spatial tasks, such as the CPP (Leal *et al*, 2014). In addition, direct injection of BDNF into the NAc has been shown to potentiate some elements of cocaine reward (Horger *et al*, 1999; Guillin *et al*, 2001; Graham *et al*, 2007). Indeed, we found that systemic injection of the BDNF agonist, 7,8-dihydroxyflavone, in naïve animals facilitated cocaine place preference (Figure 2d). This suggests that the enhanced learning effect of BDNF mediated through the hippocampus and other regions is the predominant effect. However, the present study as well as previous studies (Vargas-Perez *et al*, 2009; Koo *et al*, 2012) have shown BDNF injected directly into the VTA impairs mesolimbic DA activity and reward learning, suggesting BDNF has a varied and circuit-specific effect. How, or if, the role of BDNF in reward learning changes throughout the progression of other drug-dependent states remains to be explored, but some studies suggest the role of VTA BDNF may be altered in cocaine dependence (Lu *et al*, 2004).

We have identified a novel adaptive mechanism following chronic opioid exposure that involves microglial-BDNF-TrkB-KCC2 signaling augmenting the inhibitory GABAergic tone within the VTA. This cascade of events leads to an overall increased inhibition on VTA DA output neurons. Restoration of the Cl^−^ gradient in GABAergic neurons, either through targeting KCC2 activity or through prevention of microglial activation, may be useful new strategies for preventing and perhaps recovering from some of the reward-related behavioral deficits associated with chronic opioid use.

## FUNDING AND DISCLOSURE

We would like to acknowledge the NIDA Drug Supply Program for providing the (+) naloxone used in this study. Financial support was provided by the Shirley and Stefan Hatos Foundation (AMWT, AG; CJE is the holder of the Stefan and Shirley endowed chair), and Canadian Institutes of Health Research grant MOP 12942 (YDK) and MOP 123298 (CMC, MCO). The authors declare no conflict of interest.
