# Supplementary material for: Neuroimmune Regulation of GABAergic Neurons Within the Ventral Tegmental Area During Withdrawal from Chronic Morphine
Source: Neuropsychopharmacology. 2015 Aug 12;41(4):949–59. doi: 10.1038/npp.2015.221 (PMC4748420; doi:10.1038/npp.2015.221)
Supplement: Supplementary Information [file npp2015221x1.doc]

Supplemental Figure Legends

Supplemental Figure 1: Inhibiting microglial activation did not recover mu opioid receptor function in the VTA of opioid-dependent animals. Concentration-response curves of DAMGO stimulated [^35^S]-GTPγS binding in the VTA of opioid naïve, opioid-dependent and opioid-dependent animals treated with minocycline. E_max_ was calculated from the non-linear regression analysis performed on the above plot and compared between treatment groups. Error bars = S.E.M, **=p<0.01, n=4-6.

Supplemental Figure 2: BDNF expression in non-microglial cells of opioid-dependent animals. Withdrawal from chronic morphine leads to increased BDNF mRNA expression in the VTA. BDNF expression was co-localized with markers for DA neurons (TH) or GABAergic neurons (GAD). The increase in BDNF expression after chronic exposure to opioids was found in non-neuronal and TH+ neurons (white arrows), but rarely GABAergic neurons. Scale bar = 75uM.
