# Supplementary figures and images for: Neuroimmune Regulation of GABAergic Neurons Within the Ventral Tegmental Area During Withdrawal from Chronic Morphine
Source: Neuropsychopharmacology. 2015 Aug 12;41(4):949–59. doi: 10.1038/npp.2015.221 (PMC4748420; doi:10.1038/npp.2015.221)

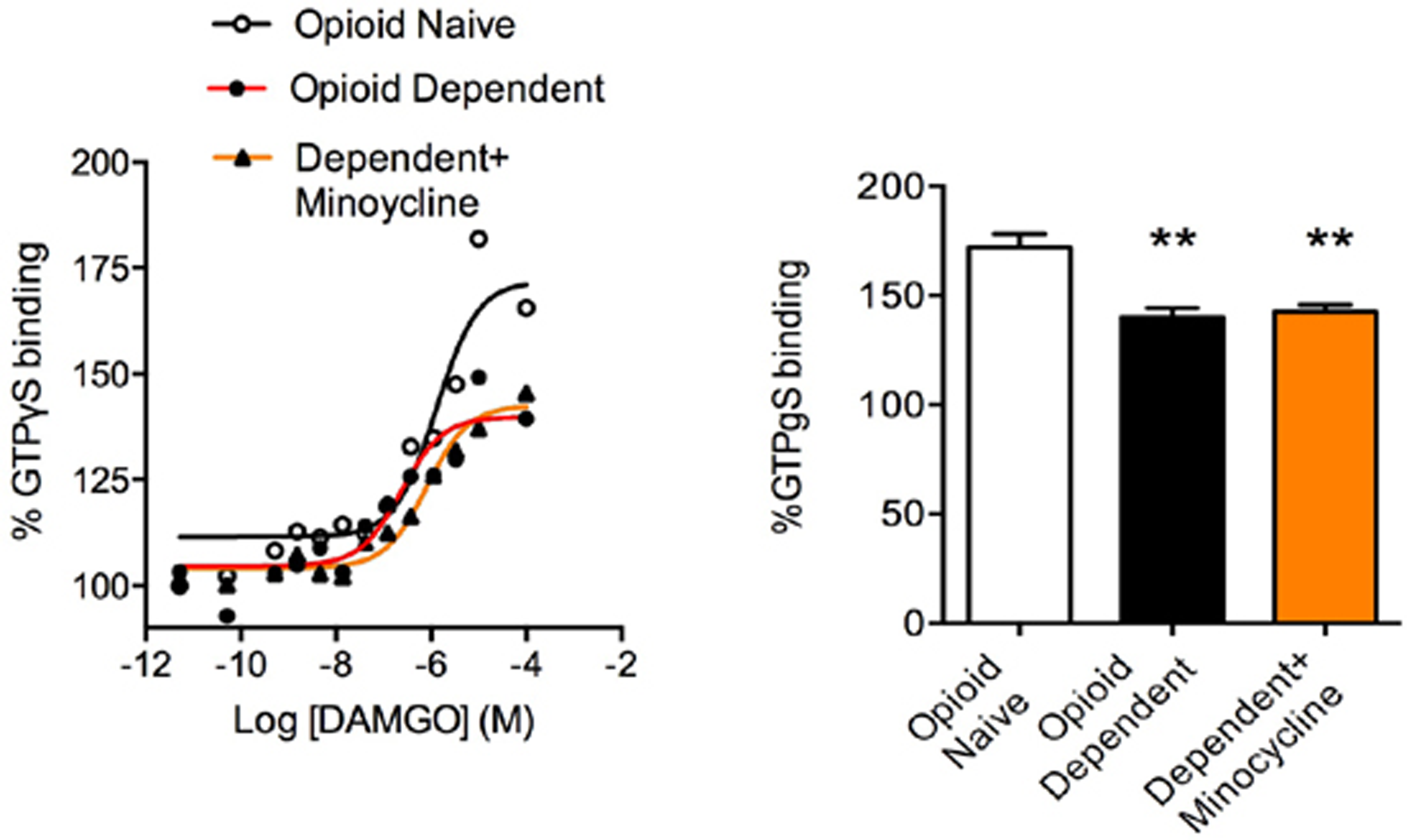

Supplement: Supplementary Figure 1 [file npp2015221x2.tif]

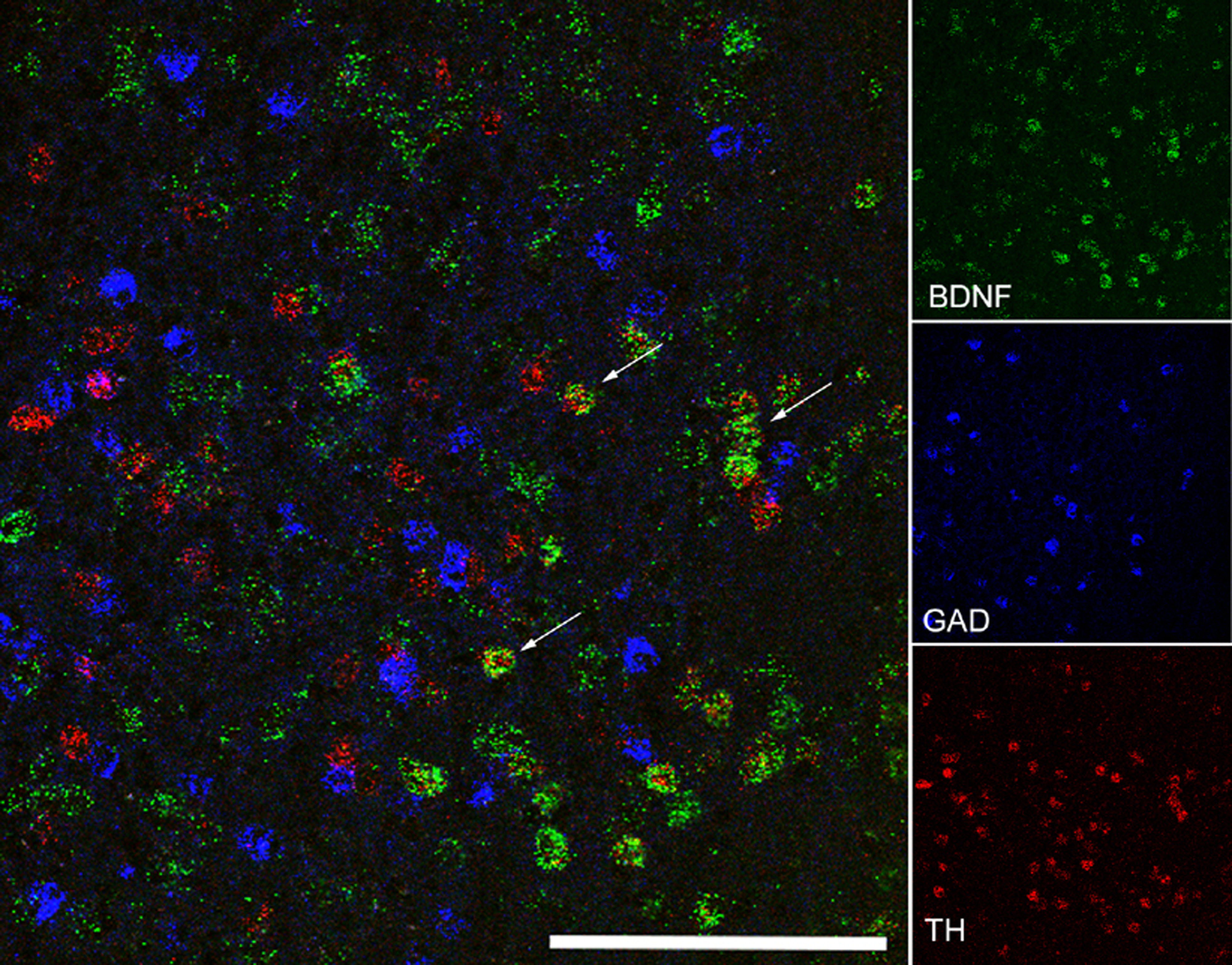

Supplement: Supplementary Figure 2 [file npp2015221x3.tif]
