# Supplementary material for: Neuroimmune Regulation of GABAergic Neurons Within the Ventral Tegmental Area During Withdrawal from Chronic Morphine
Source: Neuropsychopharmacology. 2015 Aug 12;41(4):949–59. doi: 10.1038/npp.2015.221 (PMC4748420; doi:10.1038/npp.2015.221)
Supplement: Supplementary Information [file npp2015221x4.doc]

Supplemental Material and Methods

**CPP**

*Systemic microgial inhibitor*

The conditioned place preference assay was performed using a two chamber apparatus. Each box (28x28x19cm) was divided into two equal sized conditioning chambers, separated by a guillotine door that allowed access to the two chambers. The left conditioning chamber contained vertical black and white stripes on the walls and vertical mesh bars on the floor. The right conditioning chamber contained horizontal black and white stripes on the walls and horizontal mesh bars on the floor.

Mice were randomly assigned to six groups (naïve, naïve treated with systemic minocyline or (+) naloxone, opioid dependent, and opioid dependent treated with systemic minocycline or (+) naloxone). Groups treated with microglial inhibitors received minocycline or (+) naloxone throughout the entire opioid treatment and CPP assay. During drug conditioning, animals received microglial inhibitor treatment at least 3 hours prior to drug conditioning trial. In order to counterbalance the groups before the conditioning sessions, mice were placed in the CPP apparatus and allowed free access to both the left and right chambers. The time spent in each chamber was recorded over 30 minutes using a CCD camera attached to a computer running a behavioral tracking software (Noldus Ethovision, Leesberg, VA). Groups were assigned so that any inherent preference for one side over the other was balanced between treatment groups. Animals were conditioned to cocaine or saline, and received one trial per day, totaling 8 days. The drug-paired chamber was randomly assigned to either the left or right side. During drug conditioning sessions, locomotor activity was continually monitored.

*Intra-VTA Mac-1-Saporin*

Rats were implanted with a bilateral intra-VTA cannula. Rats were used to ensure accurate targeting of the VTA, as the volume of drug required to inject could have off-target effects through diffusion in the much small mouse brain. Animals were anesthetized with isoflurane and mounted on a stereotaxic frame, and a bilateral cannula (CMA) was inserted above the VTA. After cannula implantation, animals were singly-housed and allowed to recover for 7 days. After recovery, conditioned place preference to cocaine was run. The rat CPP apparatus consisted of two large compartments of equal size (45x45x30cm) joined by a gray tunnel (18x18x30cm). The left conditioning chamber contained stripes on the walls and vertical mesh bars on the floor, whereas the right conditioning chamber contained unpainted wood on the wall and horizontal mesh grid on the floor. As before, groups were assigned to balance for any inherent bias of side in the place preference apparatus. No initial bias to either side was observed. Animals were conditioned to cocaine and saline, as above.

**Immunoblot**

Animals were anesthetized with isofluorane and the brain isolated 12 hours after the last morphine or saline injection. Brain punches (2mmx1mmx1mm) were taken from the VTA and flash frozen on dry ice. For total cell lysate preparation, punches were homogenized in cold radio-immunoprecipitation assay (RIPA) buffer with protease inhibitor on ice using a sonicator. BDNF levels were normalized to the housekeeping protein β-actin (1:5000; Sigma) and visualized with a goat anti-mouse antibody conjugated to HRP (Jackson Immunoresearch). Reactivities were visualized by an enhanced chemiluminescent detection system (Thermoscientific; Lafayette, CO).

**Immunocytochemistry**

Animals were deeply anesthetized with Pentobarbital and perfused transcardially with 4% paraformaldehyde in 0.1M phosphate buffer (PB), pH 7.4 for 10 minutes. Brains were removed from the skull, post-fixed for 30 minutes in the same fixative, and cryoprotected in 30% sucrose in 0.1M PB for 48 hours at 4^o^C. For tissue sectioning, whole brains were flash-frozen in isopentane and embedded in an optimal cutting temperature medium (Tissue Tek OCT; Sakura Finetek Europe, Alphen aan den Rijn, The Netherlands). Forty µm sections were cut at -20^o^C on a cryostat (Leica, Wetzlar, Germany) and collected in phosphate buffered saline containing 0.2% Triton X-100 (PBS-T). For KCC2 and tyrosine hydroxylase (TH) double labeling, sections were incubated overnight with the above K22C antibody and a sheep antibody against TH (1:1500, Millipore) followed by a donkey anti-rabbit IgG antibody conjugated to Alexa Fluor 594 (1:500; Invitrogen)and a donkey anti-sheep IgG antibody conjugated to Alexa Fluor 488 (1:500, Invitrogen).

For the quantification of IBA1 and KCC2 immunoreactivities, experimenters who were naïve to treatment groups of the stained sections performed the image analysis. Images were collected using a Nikon Eclipse 90i fluorescence microscope (Nikon, Tokyo, Japan) with a 20x or 40x objective. For quantitation of KCC2 immunofluorescence, images were acquired with a high-resolution digital camera with constant exposure and gain settings.

**Fluorescent *in situ* hybridization**

Animals were anesthetized with pentobarbital and perfused with 4% PFA and cryoprotected, as above. Whole brains were flash frozen in isopentane and 50uM thick sections cut onto slides on a cryostat (Leica). Sections were stored at -20oC until staining.

All sections were counterstained with DAPI. Control sections were incubated with probes for DapB, an *Escherichia coli* gene that is not expressed in mammalian cells. No labeling was detected on any control slides. Images were obtained on a laser scanning spectral confocal microscope (Leica TCS SP2) with a 63x oil-immersion objective. Two images per section (one on each side) of the VTA were collected.

**FLIM**

VTA brain slices from opioid naïve and dependent animals loaded with MQAE were transferred to a perfusion chamber (2 ml min^−1^) and extracellular MQAE was washed out for 10min in the presence of 1μM tetrodotoxin, 10μM CNQX, 40μM AP5, 0.5μM strychnine and 10μM gabazine. Furosemide (125uM) was applied to some sections to block KCC2 Cl^-^ transport. Some slices were incubated with anti-trkB (5ug/mL; Sigma) for 1 hour prior to MQAE.

GABAergic cells were visually identified as part of the VTA and could be distinguished from DA and cholinergic cells on the basis of their morphology and co-labelling in sections taken from GAD-GFP mice. Fluorescence of MQAE was acquired through a 40x water-immersion objective (0.8 NA; Zeiss, Oberkochen, Germany) and a band-pass filter (390–465nm). Fluorescence lifetime was recorded with a Becker & Hickl SPC-830 module through the non-descanned port of the Zeiss LSM510, using a band-pass filter (469/35nm, Semrock, Rochester, NY) coupled to a laser block (short-pass 750nm, Semrock). Photons were detected using a PMC-100-1 photosensor (Hamamatsu, Hamamatsu City, Japan). Experimental instrument response function of the detection path was acquired using an 80nm gold nanoparticle suspension to generate a second-harmonic signal.

For each time-point, regions of interest (ROIs) corresponding to cells were selected and added to a new phasor. Fluorescence lifetime images were acquired every 10s for a period of 7min, and the average fluorescence lifetime in each cell was extracted for each time point using a customized Matlab program (MathWorks, Natick, MA). On average, each slice contained 4.5 cells from which recordings were obtained, and averaged lifetime was expressed as the mean of all the cells/slice at each time point.

**[^35^S]GTPγS Binding Assay**

Opioid naïve and dependent mice were deeply anesthetized with isofluorane and brains isolated in ice cold PBS. The VTA was punched from 1mm-thick sections and rapidly frozen on dry ice. Tissue collected from 2 animals was pooled to obtain sufficient protein for each sample assayed. Tissue punches were homogenized in ice-cold GTPγS buffer (50mM Tris HCl, 3mM MgCl2, 0.2mM EGTA, 100mM NaCl) using a dunce homogenizer. 5 μg of protein was incubated with varying concentrations of [D-Ala^2^, N-MePhe^4^, Gly-ol^5^]-enkephalin (DAMGO) (10^-4^-10^-10.77^mM), 10mM guanosine-5’-diphosphate (GDP), and 0.1μM [^35^S]GTPγS. The well plate was incubated for 1 hour at 25^o^C, and then harvested through a dampened unifilter microplate with 1μm pore size (PerkinElmer, Waltham, MA). The filter plate was dried, filled with scintillation cocktail (Microscint, PerkinElmer) and the amount of radioactivity retained on the filter was measured by liquid scintillation spectrometry. Non-specific binding was measured in the presence of 100μM unlabeled GTPγS. Basal binding levels were measured in the absence of DAMGO. In order to compare between groups, E_max_ values were interpolated from non-linear regression analysis (sigmoidal curve fit).
